# Supplementary material for: Transcriptomic Profiling of Circular RNA in Different Brain Regions of Parkinson’s Disease in a Mouse Model
Source: Int J Mol Sci. 2020 Apr 24;21(8):3006. doi: 10.3390/ijms21083006 (PMC7216060; doi:10.3390/ijms21083006)
Supplement: Supplementary file 1 [file ijms-21-03006-s001.zip › Supplementary/Supplementary-Figure.pdf]

Figures:

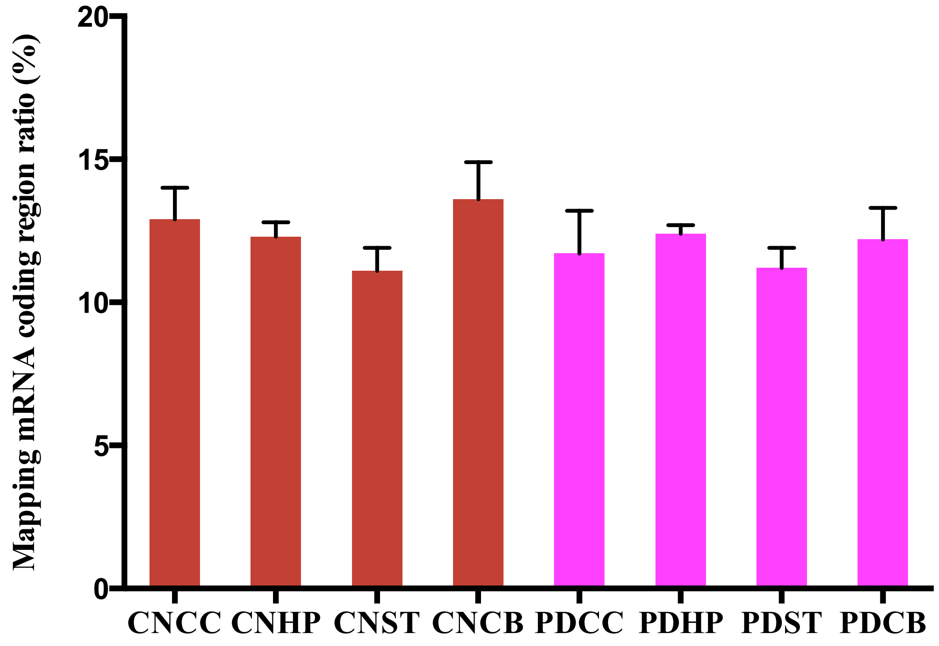

Figure S1. Mapping coding region distribution. Shows the ratio of reads mapped to the coding region in all samples.

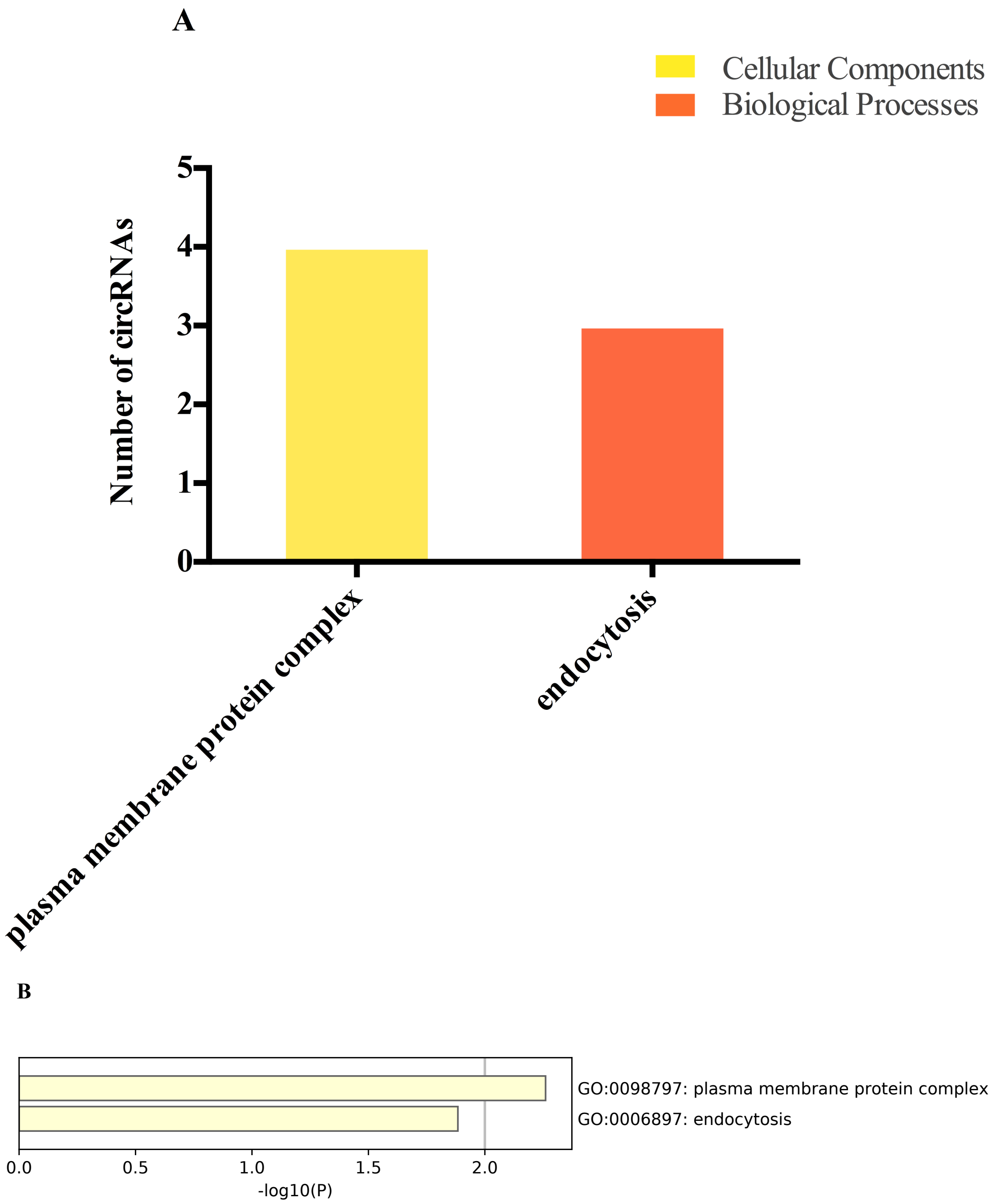

Figure. S2. GO analyses of the DE-circRNAs parental genes in the CC regions. (A) GO function classification of all DE-circRNAs in the CC region. (B) Bar chart of the all DE-circRNAs clusters in CC region.

A

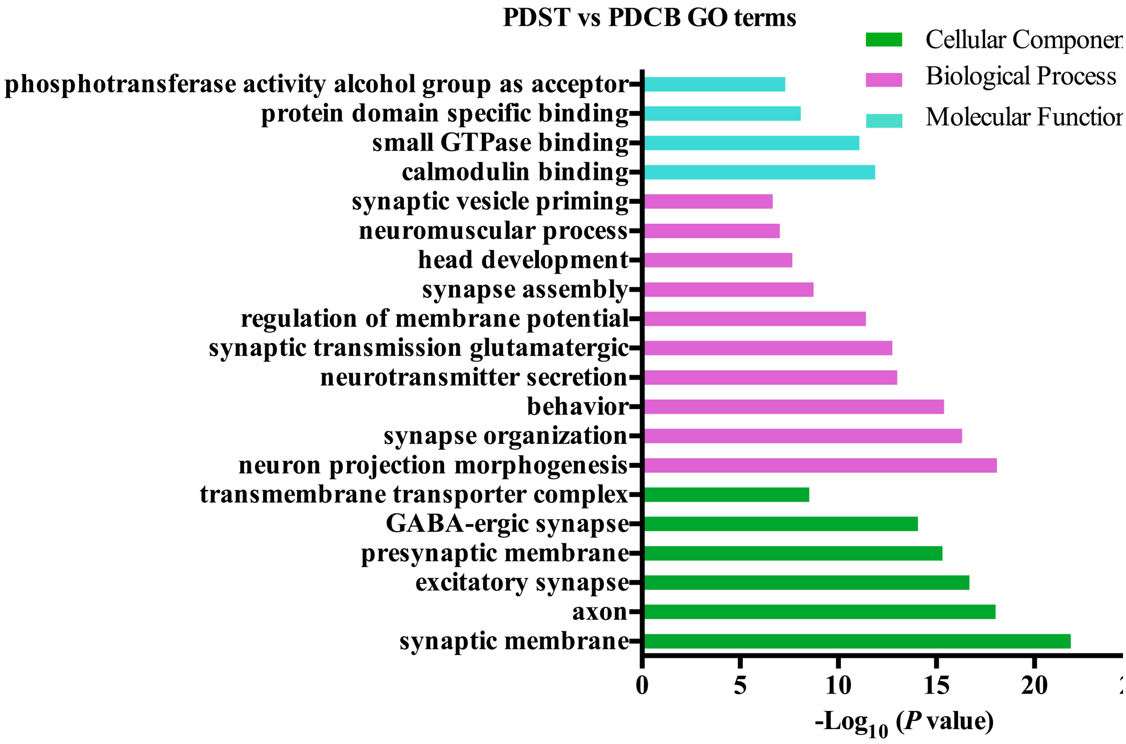
PDST vs PDCB GO terms
Cellular Componen
Biological Process
Molecular Functio
phosphotransferase activity alcohol group as acceptor
protein domain specific binding
small GTPase binding
calmodulin binding
synaptic vesicle priming
neuromuscular process
head development
synapse assembly
regulation of membrane potential
synaptic transmission glutamatergic
neurotransmitter secretion
behavior
synapse organization
neuron projection morphogenesis
transmembrane transporter complex
GABA-ergic synapse
presynaptic membrane
excitatory synapse
axon
synaptic membrane
0
5
10
15
20
-Log$_{10}$ (*P* value)

B

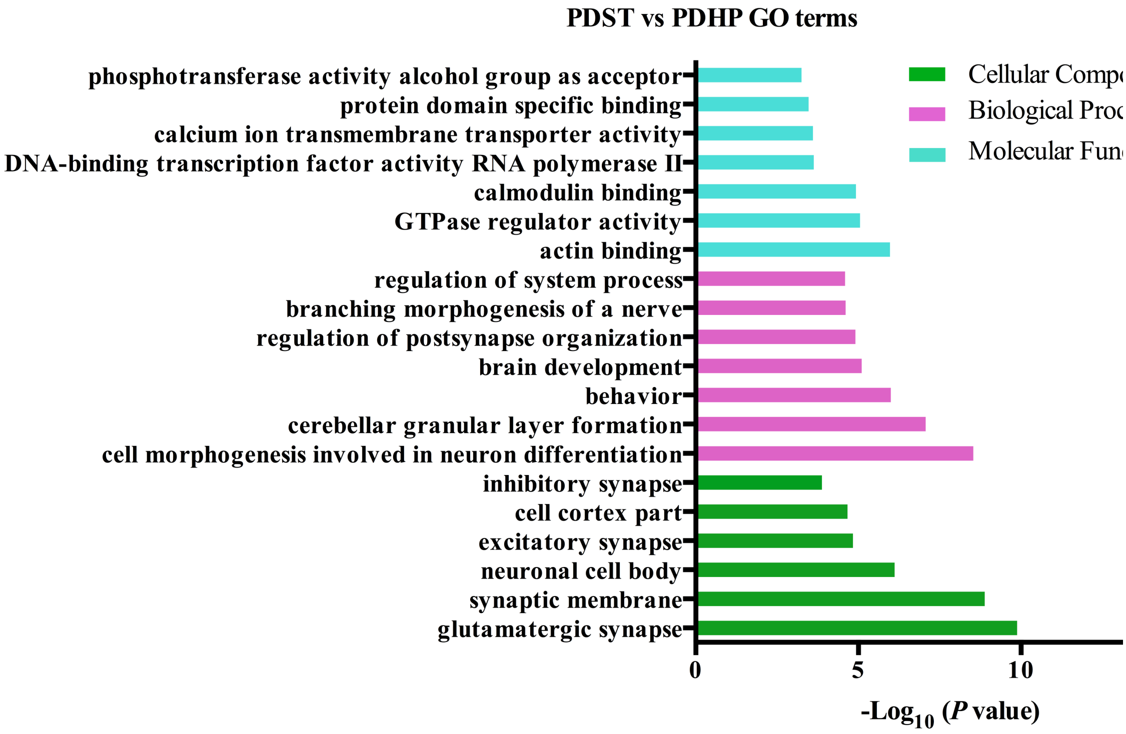
PDST vs PDHP GO terms
Cellular Comp
Biological Pro
Molecular Fun
phosphotransferase activity alcohol group as acceptor
protein domain specific binding
calcium ion transmembrane transporter activity
DNA-binding transcription factor activity RNA polymerase II
calmodulin binding
GTPase regulator activity
actin binding
regulation of system process
branching morphogenesis of a nerve
regulation of postsynapse organization
brain development
behavior
cerebellar granular layer formation
cell morphogenesis involved in neuron differentiation
inhibitory synapse
cell cortex part
excitatory synapse
neuronal cell body
synaptic membrane
glutamatergic synapse
0
5
10
-Log$_{10}$ (*P* value)

C

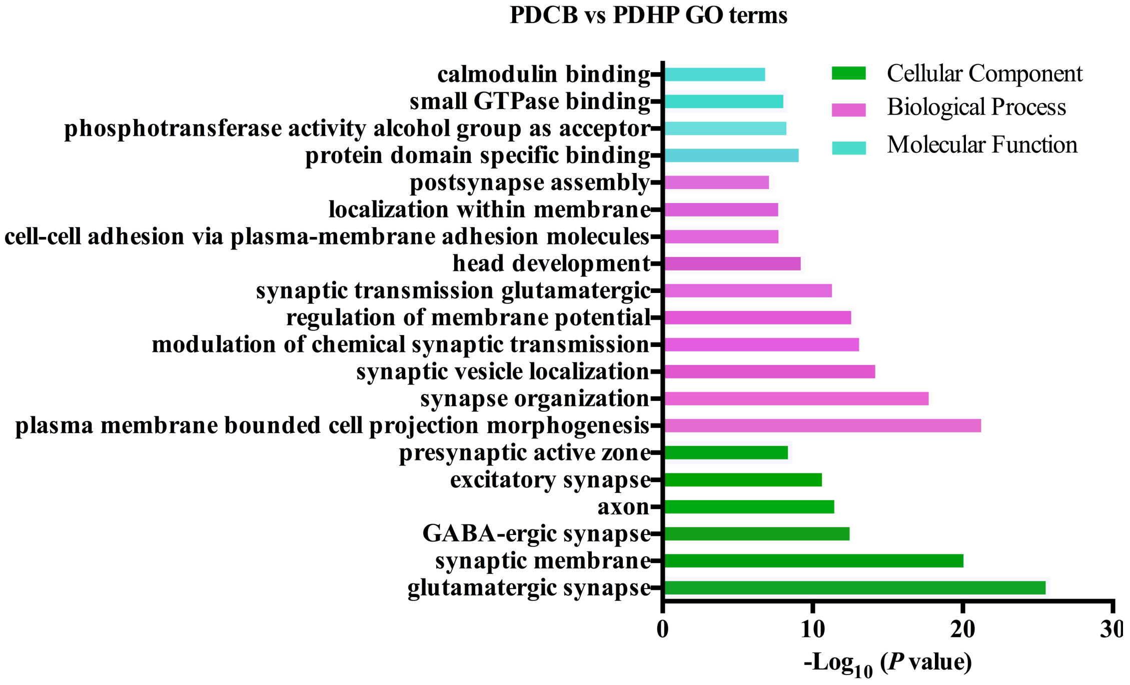
PDCB vs PDHP GO terms
calmodulin binding
small GTPase binding
phosphotransferase activity alcohol group as acceptor
protein domain specific binding
postsynapse assembly
localization within membrane
cell-cell adhesion via plasma-membrane adhesion molecules
head development
synaptic transmission glutamatergic
regulation of membrane potential
modulation of chemical synaptic transmission
synaptic vesicle localization
synapse organization
plasma membrane bounded cell projection morphogenesis
presynaptic active zone
excitatory synapse
axon
GABA-ergic synapse
synaptic membrane
glutamatergic synapse
0
10
20
30
-Log$_{10}$ (*P* value)
Cellular Component
Biological Process
Molecular Function

D

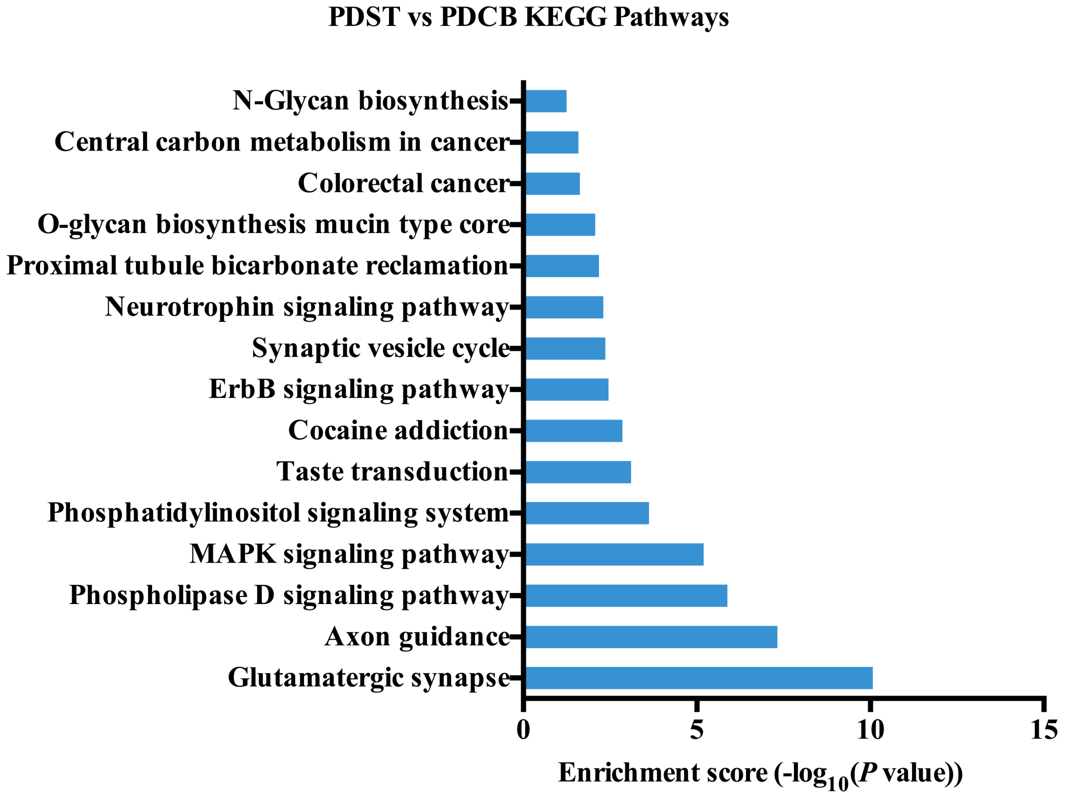
PDST vs PDCB KEGG Pathways
N-Glycan biosynthesis
Central carbon metabolism in cancer
Colorectal cancer
O-glycan biosynthesis mucin type core
Proximal tubule bicarbonate reclamation
Neurotrophin signaling pathway
Synaptic vesicle cycle
ErbB signaling pathway
Cocaine addiction
Taste transduction
Phosphatidylinositol signaling system
MAPK signaling pathway
Phospholipase D signaling pathway
Axon guidance
Glutamatergic synapse
0
5
10
15
Enrichment score (-log$_{10}$(*P* value))

E

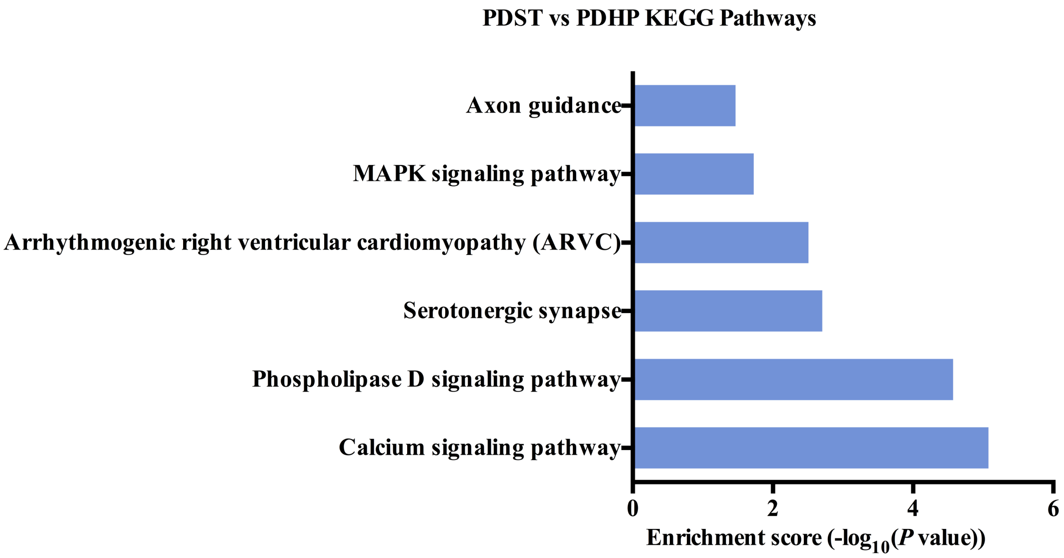

F

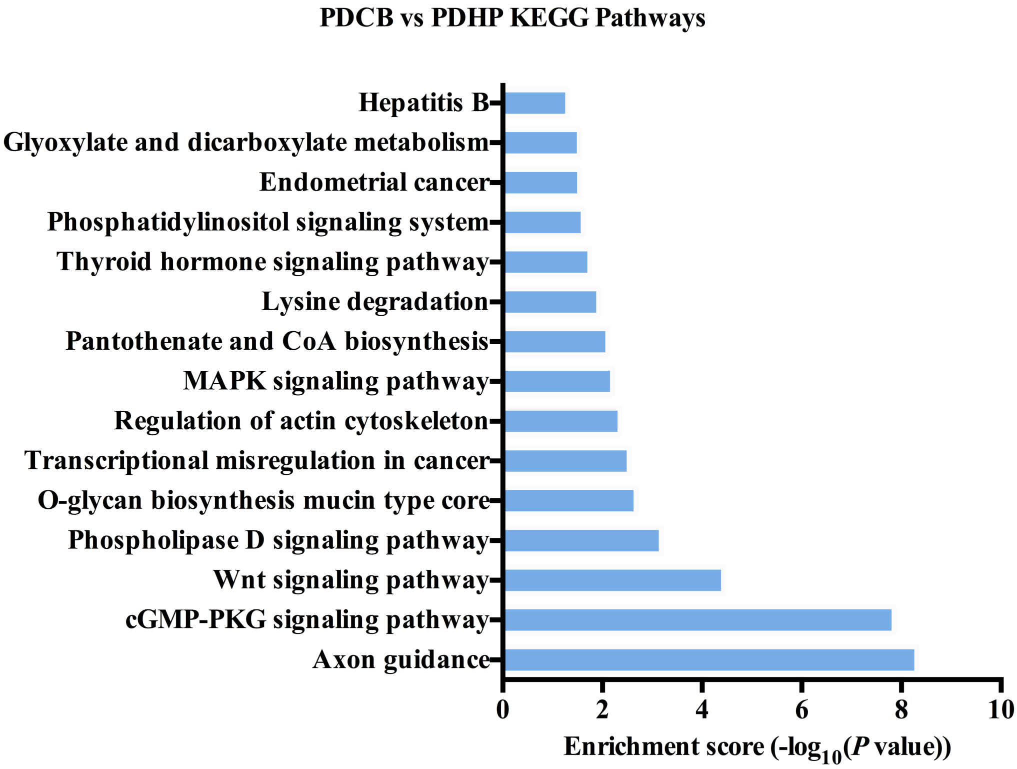

Figure S3. GO and KEGG analysis of differentially expressed circRNA host genes. (A) GO analysis was performed for the DE-circRNAs on PDST vs PDCB. (B) GO analysis was performed for the DE-circRNAs on PDST vs PDHP. (C) GO analysis was performed for the DE-circRNAs on PDCB vs PDHP. (D) KEGG analysis of the pathways of the differential circRNA parental genes on PDST vs PDCB. (E) KEGG analysis of the pathways of the differential circRNA parental genes on PDST vs PDHP. (F) KEGG analysis of the pathways of the differential circRNA parental genes on PDCB vs PDHP.

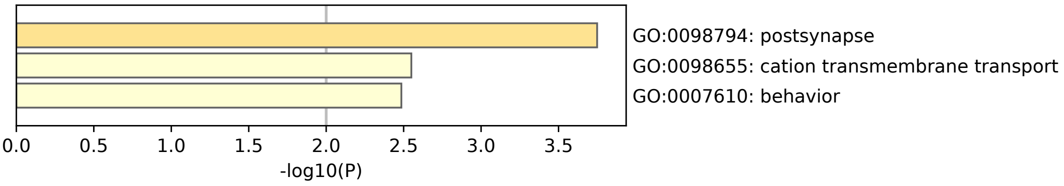

Figure. S4. GO analyses of the co-expressed DE-circRNAs parental genes in the PDST vs PDCB, PDST vs PDHP and PDCB vs PDHP groups.
