# Supplementary material for: Transcriptomic Profiling of Circular RNA in Different Brain Regions of Parkinson’s Disease in a Mouse Model
Source: Int J Mol Sci. 2020 Apr 24;21(8):3006. doi: 10.3390/ijms21083006 (PMC7216060; doi:10.3390/ijms21083006)
Supplement: Supplementary file 1 [file ijms-21-03006-s001.zip › Supplementary/Supplementary-Table.doc]

**Tables：**

Table S1 Sequencing metrics of the study.

| Sample ID | total reads | paired reads | mapped reads | mRNA reads | rRNA ratio | circRNA reads | numer exonic reads | number of circRNAs detected |
| --- | --- | --- | --- | --- | --- | --- | --- | --- |
| CNCC1 | 29,116,480 | 27,310,272 | 21,546,195 | 2,146,733 | 0.01% | 20,704 | 17,007 | 4,111 |
| CNCC2 | 34,585,942 | 32,800,597 | 24,556,018 | 2,096,123 | 0.01% | 44,898 | 40,425 | 6,837 |
| CNCC3 | 39,511,634 | 36,997,195 | 27,263,028 | 3,329,674 | 0.03% | 35,137 | 24,484 | 5,687 |
| CNHP1 | 29,497,339 | 28,280,642 | 19,173,270 | 6,99,556 | 0.02% | 34,140 | 30,145 | 5,762 |
| CNHP2 | 25,777,954 | 24,595,179 | 18,044,567 | 773,962 | 0.01% | 22,957 | 19,846 | 4,434 |
| CNHP3 | 38,356,716 | 36,419,126 | 28,000,402 | 2,126,419 | 0.02％ | 34,045 | 29,227 | 5,730 |
| CNST1 | 44,223,195 | 42,478,249 | 29,629,541 | 1,552,489 | 0.03% | 56,737 | 50,351 | 8,062 |
| CNST2 | 46,811,670 | 43,996,656 | 31,363,818 | 2,789,036 | 0.02% | 41,818 | 36,234 | 6,324 |
| CNST3 | 26,259,408 | 24,977,266 | 19,431,962 | 1,685,043 | 0.01% | 28,238 | 24,662 | 4,821 |
| CNCB1 | 40,842,710 | 39,334,459 | 27,364,615 | 1,122,983 | 0.04% | 100289 | 90,660 | 10,562 |
| CNCB2 | 31,115,899 | 29,359,403 | 22,092,288 | 1,212,319 | 0.03% | 62105 | 55,645 | 7,944 |
| CNCB3 | 29,307,526 | 28,234,938 | 21,687,569 | 718,860 | 0.02% | 86,120 | 78,340 | 9,625 |
| PDCC1 | 37,032,228 | 33,320,594 | 24,545,938 | 2,519,698 | 0.03% | 16,540 | 13,506 | 3,265 |
| PDCC2 | 23,308,933 | 21,040,565 | 15,329,238 | 2,039,219 | 0.01% | 8,734 | 6,943 | 1,941 |
| PDCC3 | 19,310,308 | 17,474,982 | 12,757,374 | 1,420,187 | 0.01% | 10,032 | 8,422 | 2,190 |
| PDHP1 | 22,602,105 | 21,226,724 | 17,425,682 | 1,252,248 | 0.03% | 19,798 | 17,003 | 3,672 |
| PDHP2 | 23,276,467 | 21,398,801 | 16,509,356 | 1,551,938 | 0.02％ | 11,979 | 9,550 | 2,573 |
| PDHP3 | 25,567,728 | 23,900,264 | 19,923,818 | 1,683,359 | 0.01% | 20,257 | 17,475 | 3,759 |
| PDST1 | 37,305,387 | 34,289,922 | 26,000,970 | 2,956,239 | 0.01% | 27,234 | 23,644 | 4,658 |
| PDST2 | 22,326,567 | 20,315,525 | 15,613,616 | 1,655,258 | 0.03% | 10,898 | 8,833 | 2,426 |
| PDST3 | 21,633,923 | 19,549,009 | 14,562,574 | 1,719,250 | 0.02% | 12,787 | 10,918 | 2,618 |
| PDCB1 | 30,326,641 | 27,916,444 | 22,527,346 | 1,940,445 | 0.02% | 41,186 | 36,619 | 5,577 |
| PDCB2 | 32,108,556 | 30,011,624 | 25,250,870 | 1,100,728 | 0.02% | 71,264 | 63,747 | 8,186 |
| PDCB3 | 21,813,651 | 20,282,055 | 17,197,720 | 907,468 | 0.03% | 44,122 | 39,088 | 5,933 |

Table S2. The significantly changed circRNAs in CC region.

| CircRNA | Chromosome | Log_2_(fold change) | CircRNA_type | Regulation | CircRNA _start | CircRNA _end | Gene | *p*-value | Gene_id |  |
| --- | --- | --- | --- | --- | --- | --- | --- | --- | --- | --- |
| chr10:127237297-127247080 | chr10 | 7.678272784 | exon | up | 127,237,297 | 127,247,080 | Kif5a | 0.0497 | ENSMUSG00000074657.5 |  |
| chr11:109011665-109012079 | chr11 | 8.891586072 | intergenic | up | 109,011,665 | 109,012,079 | None | 0.0229 | n/a |  |
| chr11:58856061-58876820 | chr11 | 6.75531513 | intergenic | up | 58,856,061 | 58,876,820 | None | 0.0002 | n/a |  |
| chr13:60896021-60935028 | chr13 | 5.076518656 | intron | up | 60,896,021 | 60,935,028 | Rasgrf2 | 0.0497 | ENSMUSG00000114487.1 |  |
| chr13:91968947-91972285 | chr13 | 5.304387363 | exon | up | 91,968,947 | 91,972,285 | Tulp4 | 0.0188 | ENSMUSG00000021708.17 |  |
| chr17:6137211-6139151 | chr17 | 7.88581374 | exon | up | 6,137,211 | 6,139,151 | None | 0.0438 | ENSMUSG00000034377.10 |  |
| chr17:70975459-70994784 | chr17 | 7.88581374 | intron | up | 70,975,459 | 70,994,784 | None | 0.0438 | ENSMUSG00000097625.1 |  |
| chr18:23583084-23602674 | chr18 | -4.26495593 | exon | down | 23,583,084 | 23,602,674 | Dtna | 0.0452 | ENSMUSG00000024302.16 |  |
| chr18:80909592-80917878 | chr18 | -4.37363655 | exon | down | 80,909,592 | 80,917,878 | Atp9b | 0.0363 | ENSMUSG00000024566.10 |  |
| chr19:29753600-29786449 | chr19 | -5.15334039 | exon | down | 29,753,600 | 29,786,449 | J03Rik | 0.0063 | ENSMUSG00000046138.15 |  |
| chr1:93835287-93978386 | chr1 | 5.113895816 | intergenic | up | 93,835,287 | 93,978,386 | None | 0.0447 | n/a |  |
| chr2:118915888-118921696 | chr2 | -4.463939046 | exon | down | 118,915,888 | 118,921,696 | Bahd1 | 0.0352 | ENSMUSG00000040007.8 |  |
| chr2:40715372-40751016 | chr2 | -4.383405824 | exon | down | 40,715,372 | 40,751,016 | Lrp1b | 0.0375 | ENSMUSG00000049252.17 |  |
| chr2:78913674-78920209 | chr2 | -4.282150479 | exon | down | 78,913,674 | 78,920,209 | Ube2e3 | 0.0449 | ENSMUSG00000027011.14 |  |
| chr3:158084456-158135409 | chr3 | 5.207778667 | exon | up | 158,084,456 | 158,135,409 | Lrrc7 | 0.0242 | ENSMUSG00000028176.12 |  |
| chr3:94697305-94937007 | chr3 | 5.610183985 | intergenic | up | 94,697,305 | 94,937,007 | None | 0.0175 | n/a |  |
| chr4:36259857-36279316 | chr4 | -4.251241574 | intron | down | 36,259,857 | 36,279,316 | Lingo2 | 0.0456 | ENSMUSG00000045083.14 |  |
| chr4:44018813-44025560 | chr4 | -4.250425041 | exon | down | 44,018,813 | 44,025,560 | Clta | 0.0461 | ENSMUSG00000028478.18 |  |
| chr5:146261244-146261318 | chr5 | -4.620915854 | intron | down | 146,261,244 | 146,261,318 | Cdk8 | 0.0217 | ENSMUSG00000029635.15 |  |
| chr7:60785638-60856304 | chr7 | 5.929907956 | intergenic | up | 60,785,638 | 60,856,304 | None | 0.0038 | n/a |  |
| chr8:20283105-20405419 | chr8 | -5.42117156 | intergenic | down | 20,283,105 | 20,405,419 | None | 0.0033 | n/a |  |
| chr8:77310731-77358634 | chr8 | -4.307872884 | exon | down | 77,310,731 | 77,358,634 | Arhgap10 | 0.0409 | ENSMUSG00000037148.8 |  |
| chr8:84864058-84864552 | chr8 | -4.614747029 | exon | down | 84,864,058 | 84,864,552 | Farsa | 0.0219 | ENSMUSG00000003808.18 |  |
| chr9:78175353-78175411 | chr9 | -3.135458764 | exon | down | 78,175,353 | 78,175,411 | Rn7sk | 0.0255 | ENSMUSG00000065037.1 |  |

Table S3. The significantly changed circRNAs in HP region.

| CircRNA | Chromosome | Log_2_(fold change) | CircRNA_type | Regulation | CircRNA_start | CircRNA_end | Gene | *p*-value | Gene_id |
| --- | --- | --- | --- | --- | --- | --- | --- | --- | --- |
| JH584304.1:25369-60857 | JH584304.1 | -1.383648008 | intergenic | down | 25,369 | 60,857 | None | 0.0268 | n/a |
| KV575234.1:213490-219686 | KV575234.1 | 1.19305786 | intergenic | up | 213,490 | 219,686 | None | 0.0353 | n/a |
| chr10:116517317-116549175 | chr10 | -2.245413077 | exon | down | 116,517,317 | 116,549,175 | Cnot2 | 0.0102 | ENSMUSG00000020166.15 |
| chr10:14004201-14066684 | chr10 | 1.005533113 | exon | up | 14,004,201 | 14,066,684 | Hivep2 | 0.0333 | ENSMUSG00000015501.10 |
| chr10:88400652-88414598 | chr10 | -4.334495306 | exon | down | 88,400,652 | 88,414,598 | Gnptab | 0.0164 | ENSMUSG00000035311.16 |
| chr10:90042546-90077142 | chr10 | -4.192479252 | exon | down | 90,042,546 | 90,077,142 | Anks1b | 0.0317 | ENSMUSG00000058589.14 |
| chr11:29848815-29906331 | chr11 | -4.124774317 | exon | down | 29,848,815 | 29,906,331 | Eml6 | 0.0370 | ENSMUSG00000044072.14 |
| chr11:53874935-53958100 | chr11 | 4.772680935 | intergenic | up | 53,874,935 | 53,958,100 | None | 0.0191 | n/a |
| chr11:85326864-85339796 | chr11 | 4.982027727 | exon | up | 85,326,864 | 85,339,796 | Ppm1d | 0.0088 | ENSMUSG00000020525.17 |
| chr12:100595815-100619641 | chr12 | -3.190710724 | exon | down | 100,595,815 | 100,619,641 | Rps6ka5 | 0.0173 | ENSMUSG00000021180.9 |
| chr12:89976347-90204818 | chr12 | -4.028492944 | exon | down | 89,976,347 | 90,204,818 | Nrxn3 | 0.0471 | ENSMUSG00000066392.11 |
| chr14:70678079-70682850 | chr14 | -4.208977212 | exon | down | 70,678,079 | 70,682,850 | Xpo7 | 0.0284 | ENSMUSG00000022100.14 |
| chr14:84477323-84477687 | chr14 | 1.621106432 | exon | up | 84,477,323 | 84,477,687 | Pcdh17 | 0.0406 | ENSMUSG00000035566.7 |
| chr16:93849130-93853898 | chr16 | -4.169257111 | exon | down | 93,849,130 | 93,853,898 | Morc3 | 0.0297 | ENSMUSG00000039456.10 |
| chr17:44638513-44658692 | chr17 | -4.137189228 | exon | down | 44,638,513 | 44,658,692 | Runx2 | 0.0328 | ENSMUSG00000039153.17 |
| chr17:63471408-63490887 | chr17 | -1.048757099 | exon | down | 63,471,408 | 63,490,887 | Fbxl17 | 0.0359 | ENSMUSG00000023965.13 |
| chr17:70975459-70994784 | chr17 | 8.656753111 | intron | up | 70,975,459 | 70,994,784 | None | 0.0000 | ENSMUSG00000097625.1 |
| chr17:7932131-8165977 | chr17 | 1.612231934 | intergenic | up | 7,932,131 | 8,165,977 | None | 0.0008 | n/a |
| chr18:10004896-10016644 | chr18 | -4.166020398 | exon | down | 10,004,896 | 10,016,644 | Usp14 | 0.0303 | ENSMUSG00000047879.17 |
| chr1:118419440-118419918 | chr1 | -4.389284004 | exon | down | 118,419,440 | 118,419,918 | Clasp1 | 0.0130 | ENSMUSG00000064302.13 |
| chr1:125568547-125579327 | chr1 | -4.073944687 | exon | down | 125,568,547 | 125,579,327 | Slc35f5 | 0.0412 | ENSMUSG00000026342.10 |
| chr1:139081173-139110485 | chr1 | 1.285072385 | exon | up | 139,081,173 | 139,110,485 | Dennd1b | 0.0278 | ENSMUSG00000056268.15 |
| chr1:140362949-140383095 | chr1 | -1.091676025 | exon | down | 140,362,949 | 140,383,095 | Kcnt2 | 0.0107 | ENSMUSG00000052726.15 |
| chr1:165604610-165608604 | chr1 | 3.463380219 | exon | up | 165,604,610 | 165,608,604 | Mpzl1 | 0.0144 | ENSMUSG00000026566.15 |
| chr1:167234692-167237648 | chr1 | -4.052634467 | exon | down | 167,234,692 | 167,237,648 | Uck2 | 0.0434 | ENSMUSG00000026558.13 |
| chr1:24711512-24731899 | chr1 | -4.639218822 | exon | down | 24,711,512 | 24,731,899 | Lmbrd1 | 0.0047 | ENSMUSG00000073725.8 |
| chr1:53885329-53914632 | chr1 | 2.434531246 | exon | up | 53,885,329 | 53,914,632 | Hecw2 | 0.0241 | ENSMUSG00000042807.15 |
| chr1:92141520-92148494 | chr1 | 2.093496011 | intron | up | 92,141,520 | 92,148,494 | Hdac4 | 0.0496 | ENSMUSG00000026313.16 |
| chr1:92148256-92148494 | chr1 | 1.687555236 | exon | up | 92,148,256 | 92,148,494 | Ryr3 | 0.0340 | ENSMUSG00000026313.16 |
| chr2:112922425-112958250 | chr2 | -4.130543684 | exon | down | 112,922,425 | 112,958,250 | Epb41l1 | 0.0352 | ENSMUSG00000057378.15 |
| chr2:156508813-156514210 | chr2 | -1.008634848 | exon | down | 156,508,813 | 156,514,210 | Acvr2a | 0.0383 | ENSMUSG00000027624.19 |
| chr2:48870289-48873473 | chr2 | -1.464414545 | exon | down | 48,870,289 | 48,873,473 | Ccdc148 | 0.0175 | ENSMUSG00000052155.5 |
| chr2:58906355-58983007 | chr2 | -4.166020398 | exon | down | 58,906,355 | 58,983,007 | Tanc1 | 0.0303 | ENSMUSG00000036641.9 |
| chr2:59646769-59724849 | chr2 | -2.883237644 | exon | down | 59,646,769 | 59,724,849 | Tlk1 | 0.0122 | ENSMUSG00000035168.16 |
| chr2:70770004-70786995 | chr2 | 1.022231675 | exon | up | 70,770,004 | 70,786,995 | Ckap5 | 0.0314 | ENSMUSG00000041997.16 |
| chr2:91555267-91560911 | chr2 | -4.292961923 | exon | down | 91,555,267 | 91,560,911 | Phf21a | 0.0189 | ENSMUSG00000040549.16 |
| chr2:92221472-92230724 | chr2 | 5.438857872 | exon | up | 92,221,472 | 92,230,724 | Sass6 | 0.0009 | ENSMUSG00000058318.15 |
| chr3:116618256-116621508 | chr3 | -4.241211316 | exon | down | 116,618,256 | 116,621,508 | Ppp3ca | 0.0234 | ENSMUSG00000027959.14 |
| chr3:136797726-136881540 | chr3 | -4.08679988 | exon | down | 136,797,726 | 136,881,540 | Nbea | 0.0393 | ENSMUSG00000028161.17 |
| chr3:55853771-55873783 | chr3 | -3.090705875 | exon | down | 55,853,771 | 55,873,783 | Rlf | 0.0241 | ENSMUSG00000027799.12 |
| chr4:121170618-121190258 | chr4 | -1.801320174 | exon | down | 121,170,618 | 121,190,258 | Zbtb48 | 0.0386 | ENSMUSG00000049878.13 |
| chr4:152020190-152020708 | chr4 | 2.662394031 | exon | up | 152,020,190 | 152,020,708 | Lmbrd1 | 0.0301 | ENSMUSG00000028952.12 |
| chr4:43087903-43115160 | chr4 | -1.615575026 | exon | down | 43,087,903 | 43,115,160 | Unc13b | 0.0409 | ENSMUSG00000028456.18 |
| chr4:82327729-82498769 | chr4 | -4.220306662 | exon | down | 82,327,729 | 82,498,769 | Nfib | 0.0248 | ENSMUSG00000008575.17 |
| chr4:9281388-9282008 | chr4 | -4.143910887 | exon | down | 9,281,388 | 9,282,008 | Clvs1 | 0.0327 | ENSMUSG00000041216.15 |
| chr5:146261244-146261318 | chr5 | -5.118103295 | intron | down | 146,261,244 | 146,261,318 | Cdk8 | 0.0006 | ENSMUSG00000029635.15 |
| chr6:116133767-116138250 | chr6 | -4.518779276 | exon | down | 116,133,767 | 116,138,250 | Tmcc1 | 0.0075 | ENSMUSG00000030126.17 |
| chr6:120870766-120876346 | chr6 | 1.643875814 | exon | up | 120,870,766 | 120,876,346 | Bcl2l13 | 0.0384 | ENSMUSG00000009112.4 |
| chr6:23287664-23323435 | chr6 | -4.159758353 | exon | down | 23,287,664 | 23,323,435 | Cadps2 | 0.0337 | ENSMUSG00000017978.18 |
| chr6:65862915-65870469 | chr6 | -4.974518947 | exon | down | 65,862,915 | 65,870,469 | Prdm5 | 0.0013 | ENSMUSG00000029913.14 |
| chr7:130739812-130753661 | chr7 | -4.143910887 | exon | down | 130,739,812 | 130,753,661 | Tacc2 | 0.0327 | ENSMUSG00000030852.17 |
| chr7:19696338-19697010 | chr7 | 2.551944441 | exon | up | 19,696,338 | 19,697,010 | Apoe | 0.0051 | ENSMUSG00000002985.16 |
| chr7:61813262-61814190 | chr7 | 2.305559945 | exon | up | 61,813,262 | 61,814,190 | Rik | 0.0075 | ENSMUSG00000109394.2 |
| chr7:81484426-81485109 | chr7 | 1.200831206 | exon | up | 81,484,426 | 81,485,109 | Ap3b2 | 0.0337 | ENSMUSG00000062444.15 |
| chr7:89279967-89307264 | chr7 | 3.482181026 | exon | up | 89,279,967 | 89,307,264 | Tmem135 | 0.0153 | ENSMUSG00000039428.11 |
| chr7:92645990-92665865 | chr7 | -1.277317042 | exon | down | 92,645,990 | 92,665,865 | Pcf11 | 0.0365 | ENSMUSG00000041328.16 |
| chr7:98610667-98637520 | chr7 | -4.365918646 | exon | down | 98,610,667 | 98,637,520 | Emsy | 0.0167 | ENSMUSG00000035401.9 |
| chr8:70102410-70104060 | chr8 | 3.304254661 | exon | up | 70,102,410 | 70,104,060 | Ncan | 0.0434 | ENSMUSG00000002341.8 |
| chr8:85875634-85922242 | chr8 | -4.186180723 | exon | down | 85,875,634 | 85,922,242 | Phkb | 0.0280 | ENSMUSG00000036879.15 |
| chr9:45972942-45978729 | chr9 | -4.436471767 | exon | down | 45,972,942 | 45,978,729 | Pafah1b2 | 0.0112 | ENSMUSG00000003131.7 |
| chr9:54383657-54404221 | chr9 | -3.558434916 | exon | down | 54,383,657 | 54,404,221 | Dmxl2 | 0.0064 | ENSMUSG00000041268.17 |
| chr9:65794622-65795495 | chr9 | 1.582097966 | exon | up | 65,794,622 | 65,795,495 | Zfp609 | 0.0037 | ENSMUSG00000040524.9 |
| chr9:72591751-72593360 | chr9 | 3.688694723 | exon | up | 72,591,751 | 72,593,360 | Rfx7 | 0.0121 | ENSMUSG00000037674.15 |
| chr9:82926397-82959851 | chr9 | -3.101763834 | exon | down | 82,926,397 | 82,959,851 | Phip | 0.0230 | ENSMUSG00000032253.13 |
| chrX:170018674-170019058 | chrX | 7.992877679 | intron | up | 170,018,674 | 170,019,058 | Erdr1 | 0.0409 | ENSMUSG00000095562.8 |
| chrY:90739142-90793680 | chrY | 1.211512069 | intergenic | up | 90,739,142 | 90,793,680 | None | 0.0492 | n/a |

Table S4. The significantly changed circRNAs in ST region.

| CircRNA | Chromosome | Log*2*(fold change) | CircRNA_type | Regulation | CircRNA_start | CircRNA_end | Gene | *p*-value | Gene_id | | |  |
| --- | --- | --- | --- | --- | --- | --- | --- | --- | --- | --- | --- | --- |
| chr10:120947204-120952255 | chr10 | 1.947274524 | exon | up | 120,947,204 | 120,952,255 | Lemd3 | 0.0366 | ENSMUSG00000048661.14 | |  | |
| chr10:123146794-123181800 | chr10 | -4.059658427 | exon | down | 123,146,794 | 123,181,800 | Usp15 | 0.0177 | ENSMUSG00000020124.10 | |  | |
| chr10:14004201-14066684 | chr10 | 1.156426147 | exon | up | 14,004,201 | 14,066,684 | Hivep2 | 0.0130 | ENSMUSG00000015501.10 | |  | |
| chr10:14004201-14086082 | chr10 | 1.429158049 | intron | up | 14,004,201 | 14,086,082 | 9330159F19Rik | 0.0458 | ENSMUSG00000015501.10 | |  | |
| chr10:29221786-29222300 | chr10 | -4.355909987 | exon | down | 29,221,786 | 29,222,300 | Anks1b | 0.0054 | ENSMUSG00000004360.9 | |  | |
| chr10:90042546-90077142 | chr10 | -2.876164084 | exon | down | 90,042,546 | 90,077,142 | Tom1l2 | 0.0393 | ENSMUSG00000058589.14 | |  | |
| chr10:90069472-90077142 | chr10 | -6.770279426 | exon | down | 90,069,472 | 90,077,142 | Arhgap44 | 0.0000 | ENSMUSG00000058589.14 | |  | |
| chr11:60270318-60275002 | chr11 | 1.175394573 | exon | up | 60,270,318 | 60,275,002 | Rhot1 | 0.0388 | ENSMUSG00000000538.18 | |  | |
| chr11:65053099-65075210 | chr11 | -4.088290551 | exon | down | 65,053,099 | 65,075,210 | Vmp1 | 0.0160 | ENSMUSG00000033389.16 | |  | |
| chr11:80223903-80227332 | chr11 | 5.305222321 | exon | up | 80,223,903 | 80,227,332 | Calm1 | 0.0019 | ENSMUSG00000017686.17 | |  | |
| chr11:86635175-86665878 | chr11 | -2.775564447 | exon | down | 86,635,175 | 86,665,878 | Lemd3 | 0.0466 | ENSMUSG00000018171.9 | |  | |
| chr12:100206156-100206233 | chr12 | 2.071799624 | exon | up | 100,206,156 | 100,206,233 | Usp15 | 0.0204 | ENSMUSG00000001175.15 | |  | |
| chr12:109718114-109718499 | chr12 | 1.67636567 | intergenic | up | 109,718,114 | 109,718,499 | None | 0.0143 | n/a | | |  |
| chr12:70733047-70759065 | chr12 | -3.848811938 | exon | down | 69,632,060 | 69,658,078 | Sos2 | 0.0357 | ENSMUSG00000034801.13 |  | | |
| chr12:8698091-8706041 | chr12 | -4.167249488 | exon | down | 8,698,091 | 8,706,041 | Pum2 | 0.0110 | ENSMUSG00000020594.14 |  | | |
| chr12:98518498-98518832 | chr12 | 4.74907486 | exon | up | 98,518,498 | 98,518,832 | Kcnk10 | 0.0368 | ENSMUSG00000033854.10 |  | | |
| chr13:17738917-17772957 | chr13 | -2.41303542 | exon | down | 17,738,917 | 17,772,957 | Cdk13 | 0.0498 | ENSMUSG00000041297.8 |  | | |
| chr13:58960530-58962916 | chr13 | -3.77665871 | exon | down | 58,859,169 | 58,861,555 | Ntrk2 | 0.0448 | ENSMUSG00000055254.15 |  | | |
| chr14:121592394-121597753 | chr14 | 3.321466213 | exon | up | 121,592,394 | 121,597,753 | Dock9 | 0.0061 | ENSMUSG00000025558.18 |  | | |
| chr14:12418005-12440915 | chr14 | 1.634412899 | exon | up | 12,418,005 | 12,440,915 | Cadps | 0.0157 | ENSMUSG00000054423.14 |  | | |
| chr14:21792404-21799284 | chr14 | 4.76570512 | intergenic | up | 21,792,404 | 21,799,284 | None | 0.0375 | n/a | | |  |
| chr14:29097784-29105025 | chr14 | 1.32200715 | exon | up | 29,097,784 | 29,105,025 | Cacna2d3 | 0.0139 | ENSMUSG00000021991.9 |  | | |
| chr15:95383626-95385156 | chr15 | 3.432904901 | exon | up | 95,383,626 | 95,385,156 | Nell2 | 0.0367 | ENSMUSG00000022454.17 |  | | |
| chr16:17378442-17389457 | chr16 | -2.838949036 | exon | down | 17,378,442 | 17,389,457 | Pi4ka | 0.0440 | ENSMUSG00000041720.14 |  | | |
| chr16:31153706-31160388 | chr16 | 1.047445996 | exon | up | 31,153,706 | 31,160,388 | Acap2 | 0.0355 | ENSMUSG00000049076.11 |  | | |
| chr16:7224311-7224550 | chr16 | 1.497927317 | exon | up | 7,224,311 | 7,224,550 | Rbfox1 | 0.0489 | ENSMUSG00000008658.16 |  | | |
| chr16:89877038-89885125 | chr16 | 2.811996857 | exon | up | 89,877,038 | 89,885,125 | Tiam1 | 0.0101 | ENSMUSG00000002489.16 |  | | |
| chr17:80044494-80053641 | chr17 | -3.991465195 | exon | down | 80,044,494 | 80,053,641 | Hnrnpll | 0.0225 | ENSMUSG00000024095.15 |  | | |
| chr17:87008803-87023913 | chr17 | 3.366312046 | exon | up | 87,008,803 | 87,023,913 | Pigf | 0.0446 | ENSMUSG00000024145.6 |  | | |
| chr17:87435089-87435148 | chr17 | -4.340682418 | exon | down | 87,435,089 | 87,435,148 | Calm2 | 0.0078 | ENSMUSG00000036438.14 |  | | |
| chr18:86461042-86473843 | chr18 | -4.089699976 | exon | down | 86,461,042 | 86,473,843 | Neto1 | 0.0163 | ENSMUSG00000050321.3 |  | | |
| chr1:128151744-128153399 | chr1 | -2.86104371 | exon | down | 128,151,744 | 128,153,399 | R3hdm1 | 0.0438 | ENSMUSG00000056211.13 |  | | |
| chr1:167234692-167237648 | chr1 | -2.671838809 | exon | down | 167,234,692 | 167,237,648 | Uck2 | 0.0410 | ENSMUSG00000026558.13 |  | | |
| chr1:185386246-185397267 | chr1 | -1.522956247 | exon | down | 185,386,246 | 185,397,267 | Eprs | 0.0415 | ENSMUSG00000026615.14 |  | | |
| chr1:192238725-192242037 | chr1 | -3.85855565 | exon | down | 192,238,725 | 192,242,037 | Kcnh1 | 0.0338 | ENSMUSG00000058248.12 |  | | |
| chr1:52708164-52709755 | chr1 | 1.496248038 | exon | up | 52,708,164 | 52,709,755 | Mfsd6 | 0.0090 | ENSMUSG00000041439.15 |  | | |
| chr2:31604614-31605343 | chr2 | 2.362023429 | exon | up | 31,604,614 | 31,605,343 | Fubp3 | 0.0205 | ENSMUSG00000026843.15 |  | | |
| chr2:70770004-70786995 | chr2 | 1.293658468 | exon | up | 70,770,004 | 70,786,995 | Tlk1 | 0.0002 | ENSMUSG00000041997.16 |  | | |
| chr3:29148347-29153601 | chr3 | -1.022269303 | exon | down | 29,148,347 | 2,915,3601 | Egfem1 | 0.0377 | ENSMUSG00000063600.14 |  | | |
| chr3:55853771-55955493 | chr3 | -4.290229042 | exon | down | 55,853,771 | 55,955,493 | Nbea | 0.0071 | ENSMUSG00000027799.12 |  | | |
| chr4:130327193-130338584 | chr4 | -3.971840963 | exon | down | 130,327,193 | 130,338,584 | Zcchc17 | 0.0230 | ENSMUSG00000028772.19 |  | | |
| chr4:150431523-150439468 | chr4 | 5.612833917 | exon | up | 150,431,523 | 150,439,468 | Rere | 0.0004 | ENSMUSG00000039852.17 |  | | |
| chr4:151579701-151586482 | chr4 | 1.986653339 | intron | up | 151,579,701 | 151,586,482 | Camta1 | 0.0256 | ENSMUSG00000014592.20 |  | | |
| chr4:48131765-48140485 | chr4 | -3.774333917 | exon | down | 48,131,765 | 48,140,485 | Stx17 | 0.0456 | ENSMUSG00000061455.13 |  | | |
| chr4:99212577-99235186 | chr4 | -2.850130163 | exon | down | 99,212,577 | 99,235,186 | Atg4c | 0.0382 | ENSMUSG00000028550.15 |  | | |
| chr5:110753804-110756766 | chr5 | -4.077980568 | exon | down | 110,753,804 | 110,756,766 | Ep400 | 0.0154 | ENSMUSG00000029505.17 |  | | |
| chr5:127789723-127792622 | chr5 | -3.984757616 | exon | down | 127,789,723 | 127,792,622 | Tmem132d | 0.0214 | ENSMUSG00000034310.8 |  | | |
| chr5:130101590-130102535 | chr5 | -3.863085434 | exon | down | 130,101,590 | 130,102,535 | Tpst1 | 0.0330 | ENSMUSG00000034118.15 |  | | |
| chr5:15805747-15833862 | chr5 | 5.553452198 | exon | up | 16,264,258 | 16,267,475 | Cacna2d1 | 0.0005 | ENSMUSG00000040118.15 |  | | |
| chr5:27382755-27399012 | chr5 | 1.77985667 | exon | up | 27,382,755 | 27,399,012 | Dpp6 | 0.0141 | ENSMUSG00000061576.15 |  | | |
| chr5:43165919-43191404 | chr5 | -2.469769303 | exon | down | 43,165,919 | 43,191,404 | Gm7854 | 0.0429 | ENSMUSG00000085720.8 |  | | |
| chr5:96528501-96545083 | chr5 | -3.881957464 | exon | down | 96,528,501 | 96,545,083 | Fras1 | 0.0362 | ENSMUSG00000034687.8 |  | | |
| chr6:135772193-135780336 | chr6 | -1.586584804 | exon | down | 135,772,193 | 135,780,336 | Grin2b | 0.0047 | ENSMUSG00000030209.14 |  | | |
| chr6:36815791-36887400 | chr6 | -3.855931685 | exon | down | 36,865,791 | 36,937,400 | Dgki | 0.0345 | ENSMUSG00000038665.15 |  | | |
| chr6:39506701-39508369 | chr6 | 3.818190609 | exon | up | 39,506,701 | 39,508,369 | Dennd2a | 0.0072 | ENSMUSG00000038456.9 |  | | |
| chr6:52619977-52630349 | chr6 | -2.580863246 | exon | down | 52,619,977 | 52,630,349 | Hibadh | 0.0116 | ENSMUSG00000029776.11 |  | | |
| chr6:86710515-86711679 | chr6 | 1.6886523 | exon | up | 86,710,515 | 86,711,679 | Gmcl1 | 0.0408 | ENSMUSG00000001157.13 |  | | |
| chr7:124029586-124029805 | chr7 | 2.43806685 | intron | up | 124,029,586 | 124,029,805 | Hs3st4 | 0.0211 | ENSMUSG00000078591.1 |  | | |
| chr7:19696338-19697010 | chr7 | 2.649131016 | exon | up | 19,696,338 | 19,697,010 | Apoe | 0.0075 | ENSMUSG00000002985.16 |  | | |
| chr7:46632660-46635556 | chr7 | -3.810340463 | exon | down | 46,632,660 | 46,635,556 | Sergef | 0.0460 | ENSMUSG00000030839.12 |  | | |
| chr7:59352999-59432670 | chr7 | 4.76570512 | intron | up | 59,352,999 | 59,432,670 | Ipw | 0.0375 | ENSMUSG00000100826.6 |  | | |
| chr7:59405045-59405592 | chr7 | -5.009433403 | exon | down | 59,405,045 | 59,405,592 | Otud7a | 0.0004 | ENSMUSG00000100826.6 |  | | |
| chr7:63649609-63650965 | chr7 | 3.496152982 | exon | up | 63,649,609 | 63,650,965 | Gm7854 | 0.0207 | ENSMUSG00000033510.14 |  | | |
| chr8:55037328-55055441 | chr8 | -4.140949358 | exon | down | 55,037,328 | 55,055,441 | Gpm6a | 0.0136 | ENSMUSG00000031517.8 |  | | |
| chr8:58427700-58536014 | chr8 | -2.466611418 | exon | down | 58,427,700 | 58,536,014 | Galntl6 | 0.0427 | ENSMUSG00000096914.3 |  | | |
| chr8:69659263-69659634 | chr8 | 2.759432559 | exon | up | 69,659,263 | 6,9659,634 | Zfp964 | 0.0102 | ENSMUSG00000091764.3 |  | | |
| chr8:88705226-88719396 | chr8 | -4.01905323 | exon | down | 88,705,226 | 88,719,396 | Cyld | 0.0188 | ENSMUSG00000036712.15 |  | | |
| chr9:104989572-104993887 | chr9 | 3.066824509 | exon | up | 104,989,572 | 104,993,887 | Cpne4 | 0.0365 | ENSMUSG00000032564.16 |  | | |
| chr9:72591751-72593360 | chr9 | 1.502445131 | exon | up | 72,591,751 | 72,593,360 | Rfx7 | 0.0489 | ENSMUSG00000037674.15 |  | | |
| chr9:75116186-75123055 | chr9 | -1.668379348 | exon | down | 75,116,186 | 75,123,055 | Myo5a | 0.0316 | ENSMUSG00000034593.16 |  | | |
| chrY:90739142-90793680 | chrY | 1.123358047 | intergenic | up | 90739142 | 90793680 | None | 0.0139 | n/a | | |  |

Table S5. The significantly changed circRNAs in CB region.

| CircRNA | Chromosome | Log_2_(fold change) | CircRNA_type | Regulation | CircRNA_start | CircRNA_end | Gene | *p*-value | Gene_id |
| --- | --- | --- | --- | --- | --- | --- | --- | --- | --- |
| KV575237.1:932636-932848 | KV575237.1 | 2.915109264 | intergenic | up | 932,636 | 932,848 | None | 0.0334 | n/a |
| chr10:120825108-120852092 | chr10 | 2.520816287 | exon | up | 120,825,108 | 120,852,092 | Msrb3 | 0.0454 | ENSMUSG00000051236.13 |
| chr10:121925816-121931509 | chr10 | -1.657523001 | exon | down | 121,925,816 | 121,931,509 | Srgap1 | 0.0385 | ENSMUSG00000020121.15 |
| chr10:12421597-12455564 | chr10 | 4.569492581 | exon | up | 12,421,597 | 12,455,564 | Utrn | 0.0293 | ENSMUSG00000019820.11 |
| chr10:128358334-128358533 | chr10 | 1.590793174 | exon | up | 128,358,334 | 128,358,533 | Cs | 0.0219 | ENSMUSG00000005683.8 |
| chr10:13801404-13801949 | chr10 | -1.300268281 | exon | down | 13,801,404 | 13,801,949 | Aig1 | 0.0135 | ENSMUSG00000019806.13 |
| chr10:29196265-29199280 | chr10 | 2.763246721 | exon | up | 29,196,265 | 29,199,280 | Soga3 | 0.0137 | ENSMUSG00000038916.7 |
| chr10:42196466-42197901 | chr10 | -1.634732342 | exon | down | 42,196,466 | 42,197,901 | Foxo3 | 0.0230 | ENSMUSG00000048756.11 |
| chr10:62832894-62840374 | chr10 | -4.210268028 | exon | down | 62,832,894 | 62,840,374 | Tet1 | 0.0232 | ENSMUSG00000047146.17 |
| chr10:67034599-67063071 | chr10 | -4.106289775 | exon | down | 67,034,599 | 67,063,071 | Reep3 | 0.0345 | ENSMUSG00000019873.8 |
| chr10:7126095-7154485 | chr10 | -2.87141963 | exon | down | 7,126,095 | 7,154,485 | Cnksr3 | 0.0337 | ENSMUSG00000015202.13 |
| chr11:116262860-116263297 | chr11 | 1.162851773 | exon | up | 116,262,860 | 116,263,297 | Srp68 | 0.0344 | ENSMUSG00000020780.15 |
| chr11:53874935-53958100 | chr11 | 5.313051664 | intergenic | up | 53,874,935 | 53,958,100 | None | 0.0009 | n/a |
| chr11:65057297-65075210 | chr11 | -4.143540812 | exon | down | 65,057,297 | 65,075,210 | Arhgap44 | 0.0312 | ENSMUSG00000033389.16 |
| chr11:72459039-72476675 | chr11 | 2.871447229 | exon | up | 72,645,537 | 72,663,173 | Ube2g1 | 0.0384 | ENSMUSG00000020794.13 |
| chr11:93905159-93933958 | chr11 | -4.270496247 | exon | down | 93,905,159 | 93,933,958 | Mbtd1 | 0.0192 | ENSMUSG00000059474.13 |
| chr11:97229830-97238258 | chr11 | -1.570312595 | exon | down | 97,229,830 | 97,238,258 | Npepps | 0.0022 | ENSMUSG00000001441.13 |
| chr11:99321216-99323680 | chr11 | -4.309389275 | intergenic | down | 99,321,216 | 99,323,680 | None | 0.0163 | n/a |
| chr12:31760847-31839854 | chr12 | -4.82594255 | exon | down | 31,760,847 | 31,839,854 | Cog5 | 0.0017 | ENSMUSG00000035933.5 |
| chr12:3749470-3751907 | chr12 | 1.73086018 | exon | up | 3,749,470 | 3,751,907 | Dtnb | 0.0439 | ENSMUSG00000071454.13 |
| chr12:55130616-55199786 | chr12 | 6.803602449 | intergenic | up | 55,130,616 | 55,199,786 | None | 0.0000 | n/a |
| chr13:104792499-104797343 | chr13 | 1.545528806 | exon | up | 104,792,499 | 104,797,343 | Cwc27 | 0.0394 | ENSMUSG00000021715.12 |
| chr13:59473688-59482604 | chr13 | -2.922817495 | exon | down | 59,473,688 | 59,482,604 | Agtpbp1 | 0.0294 | ENSMUSG00000021557.15 |
| chr13:69510620-69515903 | chr13 | -4.270496247 | exon | down | 69,510,620 | 69,515,903 | Tent4a | 0.0192 | ENSMUSG00000034575.13 |
| chr14:103174766-103177330 | chr14 | -4.302024297 | exon | down | 103,174,766 | 103,177,330 | Mycbp2 | 0.0169 | ENSMUSG00000033004.15 |
| chr14:122133280-122139774 | chr14 | -1.783820732 | exon | down | 122,133,280 | 122,139,774 | Tm9sf2 | 0.0299 | ENSMUSG00000025544.13 |
| chr14:123507481-123515773 | chr14 | 3.231432917 | exon | up | 123,507,481 | 123,515,773 | Nalcn | 0.0357 | ENSMUSG00000000197.8 |
| chr14:13995007-14053023 | chr14 | 4.702905296 | exon | up | 13,995,007 | 14,053,023 | Atxn7 | 0.0178 | ENSMUSG00000021738.5 |
| chr14:25502655-25536824 | chr14 | 1.496037093 | exon | up | 25,502,655 | 25,536,824 | Zmiz1 | 0.0152 | ENSMUSG00000007817.15 |
| chr14:67443182-67448819 | chr14 | -4.102337165 | exon | down | 66,824,345 | 66,829,982 | Dpysl2 | 0.0321 | ENSMUSG00000022048.8 |
| chr15:100955410-100957589 | chr15 | -4.587785114 | exon | down | 100,955,410 | 100,957,589 | Scn8a | 0.0045 | ENSMUSG00000023033.14 |
| chr15:40752896-40870646 | chr15 | -4.362102953 | exon | down | 40,752,896 | 40,870,646 | Zfpm2 | 0.0133 | ENSMUSG00000022306.9 |
| chr15:79114655-79115171 | chr15 | 1.60797957 | exon | up | 79,114,655 | 79,115,171 | Micall1 | 0.0424 | ENSMUSG00000033039.10 |
| chr15:79536997-79537545 | chr15 | 1.809170199 | exon | up | 79,536,997 | 79,537,545 | Ddx17 | 0.0387 | ENSMUSG00000055065.7 |
| chr15:8147143-8148451 | chr15 | -4.166872982 | exon | down | 8,147,143 | 8,148,451 | Nup155 | 0.0261 | ENSMUSG00000022142.8 |
| chr15:91595063-91610440 | chr15 | -4.005683534 | exon | down | 91,764,632 | 91,780,009 | Lrrk2 | 0.0448 | ENSMUSG00000036273.15 |
| chr16:13687391-13688925 | chr16 | -3.060282303 | exon | down | 13,687,391 | 13,688,925 | Bfar | 0.0318 | ENSMUSG00000022684.15 |
| chr16:14419700-14423260 | chr16 | -2.688248853 | exon | down | 14,419,700 | 14,423,260 | Abcc1 | 0.0137 | ENSMUSG00000023088.17 |
| chr16:32950292-32959531 | chr16 | -4.229256732 | exon | down | 32,950,292 | 32,959,531 | Lrch3 | 0.0199 | ENSMUSG00000022801.13 |
| chr16:32952057-32961744 | chr16 | -4.076156553 | exon | down | 32,952,057 | 32,961,744 | Zbtb11 | 0.0363 | ENSMUSG00000022801.13 |
| chr16:55980595-55982406 | chr16 | -4.267989729 | exon | down | 55,980,595 | 55,982,406 | Rbfox1 | 0.0181 | ENSMUSG00000022601.11 |
| chr16:6809207-6809248 | chr16 | 4.431298008 | exon | up | 6,809,207 | 6,809,248 | Pkd1 | 0.0466 | ENSMUSG00000008658.16 |
| chr16:7224311-7224550 | chr16 | 1.153350963 | exon | up | 7,224,311 | 7,224,550 | Gnptg | 0.0253 | ENSMUSG00000008658.16 |
| chr17:24577750-24578029 | chr17 | 2.890124918 | exon | up | 24,577,750 | 24,578,029 | Wdr36 | 0.0388 | ENSMUSG00000032855.6 |
| chr17:25234808-25235111 | chr17 | 3.667522425 | exon | up | 25,234,808 | 25,235,111 | Arhgap26 | 0.0085 | ENSMUSG00000035521.13 |
| chr18:32861027-32863262 | chr18 | -4.154744514 | exon | down | 32,861,027 | 32,863,262 | Hmgxb3 | 0.0294 | ENSMUSG00000038299.16 |
| chr18:39126833-39205320 | chr18 | -4.390910832 | exon | down | 39,126,833 | 39,205,320 | Wdr7 | 0.0105 | ENSMUSG00000036452.18 |
| chr18:61140791-61141790 | chr18 | -1.039677835 | exon | down | 61,140,791 | 61,141,790 | Micall1 | 0.0451 | ENSMUSG00000024622.8 |
| chr18:63755036-63760785 | chr18 | -1.246519017 | exon | down | 63,755,036 | 63,760,785 | Ddx17 | 0.0102 | ENSMUSG00000040560.10 |
| chr18:69564034-69564684 | chr18 | 2.75568191 | exon | up | 69,564,034 | 69,564,684 | Tcf4 | 0.0016 | ENSMUSG00000053477.17 |
| chr19:22697422-22793454 | chr19 | 1.625335482 | exon | up | 22,697,422 | 22,793,454 | Trpm3 | 0.0466 | ENSMUSG00000052387.16 |
| chr19:34840757-34841109 | chr19 | -1.803223281 | exon | down | 34,840,757 | 34,841,109 | Pank1 | 0.0478 | ENSMUSG00000033610.16 |
| chr19:5801885-5801966 | chr19 | 5.145671604 | exon | up | 5,801,885 | 5,801,966 | Malat1 | 0.0023 | ENSMUSG00000092341.3 |
| chr1:139040000-139042043 | chr1 | 1.637920095 | exon | up | 139,040,000 | 139,042,043 | Dennd1b | 0.0224 | ENSMUSG00000056268.15 |
| chr1:160627215-160645401 | chr1 | -2.916073006 | exon | down | 160,627,215 | 160,645,401 | Rabgap1l | 0.0298 | ENSMUSG00000026721.16 |
| chr1:24085411-24086611 | chr1 | 1.523608709 | exon | up | 24,085,411 | 24,086,611 | Fam135a | 0.0447 | ENSMUSG00000026153.15 |
| chr1:24559654-24575459 | chr1 | -3.091075763 | exon | down | 24,559,654 | 24,575,459 | Col19a1 | 0.0282 | ENSMUSG00000026141.13 |
| chr1:25420558-25460811 | chr1 | -2.678705431 | exon | down | 25,420,558 | 25,460,811 | Adgrb3 | 0.0337 | ENSMUSG00000033569.17 |
| chr1:38107568-38108467 | chr1 | 1.381373245 | exon | up | 38,107,568 | 38,108,467 | Rev1 | 0.0069 | ENSMUSG00000026082.11 |
| chr1:64078707-64079334 | chr1 | 1.253086553 | exon | up | 64,078,707 | 64,079,334 | Klf7 | 0.0267 | ENSMUSG00000025959.13 |
| chr1:92148256-92148494 | chr1 | 1.108707932 | exon | up | 92,148,256 | 92,148,494 | Hdac4 | 0.0142 | ENSMUSG00000026313.16 |
| chr2:113426522-113444459 | chr2 | -3.975433621 | intron | down | 113,426,522 | 113,444,459 | Fmn1 | 0.0498 | ENSMUSG00000044042.18 |
| chr2:120806759-120825315 | chr2 | -5.118253332 | exon | down | 120,806,759 | 120,825,315 | Ttbk2 | 0.0006 | ENSMUSG00000090100.7 |
| chr2:125767375-125768333 | chr2 | -4.181814654 | exon | down | 125,767,375 | 125,768,333 | Secisbp2l | 0.0241 | ENSMUSG00000035093.16 |
| chr2:139910533-139951579 | chr2 | -4.305403986 | exon | down | 139,910,533 | 139,951,579 | Tasp1 | 0.0156 | ENSMUSG00000039033.11 |
| chr2:139947157-140057499 | chr2 | -4.468058976 | exon | down | 139,947,157 | 140,057,499 | Tasp1 | 0.0073 | ENSMUSG00000039033.11 |
| chr2:139947170-139951579 | chr2 | 2.028350886 | exon | up | 139,947,170 | 139,951,579 | Tasp1 | 0.0144 | ENSMUSG00000039033.11 |
| chr2:139996073-140057499 | chr2 | -1.733114625 | exon | down | 139,996,073 | 140,057,499 | Tasp1 | 0.0271 | ENSMUSG00000039033.11 |
| chr2:142739523-142740991 | chr2 | 2.967601451 | exon | up | 142,739,523 | 142,740,991 | Kif16b | 0.0008 | ENSMUSG00000038844.10 |
| chr2:153194277-153195565 | chr2 | -2.957910197 | exon | down | 153,194,277 | 153,195,565 | Tm9sf4 | 0.0415 | ENSMUSG00000068040.10 |
| chr2:31604614-31605343 | chr2 | 1.701718945 | exon | up | 31,604,614 | 31,605,343 | Fubp3 | 0.0057 | ENSMUSG00000026843.15 |
| chr3:123546769-123562079 | chr3 | -4.465522792 | exon | down | 123,546,769 | 123,562,079 | Ndst3 | 0.0078 | ENSMUSG00000027977.15 |
| chr3:123627959-123672477 | chr3 | -4.018418838 | exon | down | 123,627,959 | 123,672,477 | Ank2 | 0.0428 | ENSMUSG00000027977.15 |
| chr3:127048182-127057091 | chr3 | -4.447299793 | exon | down | 127,048,182 | 127,057,091 | Tut4 | 0.0080 | ENSMUSG00000032826.18 |
| chr4:108478873-108491464 | chr4 | -3.992809544 | exon | down | 108,478,873 | 108,491,464 | Frg2f1 | 0.0478 | ENSMUSG00000034610.14 |
| chr4:119533313-119533809 | chr4 | -4.335382223 | exon | down | 119,533,313 | 119,533,809 | Prdm2 | 0.0130 | ENSMUSG00000087385.8 |
| chr4:143170036-143194734 | chr4 | -4.415488703 | exon | down | 143,170,036 | 143,194,734 | Zcchc7 | 0.0096 | ENSMUSG00000057637.13 |
| chr4:44893533-44895989 | chr4 | -2.07109017 | exon | down | 44,893,533 | 44,895,989 | Astn2 | 0.0410 | ENSMUSG00000035649.17 |
| chr4:65911660-65992434 | chr4 | 4.913367417 | exon | up | 65,911,660 | 65,992,434 | Ptprd | 0.0071 | ENSMUSG00000028373.16 |
| chr4:76039281-76059829 | chr4 | -3.020668486 | exon | down | 76,039,281 | 76,059,829 | Zfp644 | 0.0345 | ENSMUSG00000028399.18 |
| chr5:106619540-106666845 | chr5 | -2.865467641 | exon | down | 106,619,540 | 106,666,845 | Gtf2ird1 | 0.0377 | ENSMUSG00000049606.16 |
| chr5:106634831-106690877 | chr5 | 4.695054213 | exon | up | 106,634,831 | 106,690,877 | Limk1 | 0.0195 | ENSMUSG00000049606.16 |
| chr5:134360954-134362548 | chr5 | 5.145373968 | exon | up | 134,360,954 | 134,362,548 | Gnai1 | 0.0022 | ENSMUSG00000023079.14 |
| chr5:134664185-134666013 | chr5 | -4.076156553 | exon | down | 134,664,185 | 134,666,013 | Magi2 | 0.0363 | ENSMUSG00000029674.13 |
| chr5:17814371-17814643 | chr5 | 1.074004443 | exon | up | 18,308,553 | 18,308,825 | Zfp512 | 0.0431 | ENSMUSG00000057614.6 |
| chr5:20465576-20466214 | chr5 | -2.298495319 | exon | down | 20,465,576 | 20,466,214 | Dcun1d4 | 0.0271 | ENSMUSG00000040003.18 |
| chr5:31466232-31468240 | chr5 | 1.575256851 | exon | up | 31,466,232 | 31,468,240 | Fubp3 | 0.0071 | ENSMUSG00000062761.7 |
| chr5:73520116-73531574 | chr5 | -4.393704883 | exon | down | 73,520,116 | 73,531,574 | Ndst3 | 0.0101 | ENSMUSG00000051674.15 |
| chr5:90287249-90298134 | chr5 | -2.003745565 | exon | down | 90,287,249 | 90,298,134 | Ankrd17 | 0.0259 | ENSMUSG00000055204.15 |
| chr6:104701162-104728368 | chr6 | -1.205565833 | exon | down | 104,701,162 | 104,728,368 | Cntn6 | 0.0135 | ENSMUSG00000030092.14 |
| chr6:119002577-119005081 | chr6 | 3.481296938 | intron | up | 119,002,577 | 119,005,081 | Cacna1c | 0.0038 | ENSMUSG00000051331.15 |
| chr6:120870766-120876346 | chr6 | 1.53276727 | exon | up | 120,870,766 | 120,876,346 | Bcl2l13 | 0.0060 | ENSMUSG00000009112.4 |
| chr6:31139729-31181149 | chr6 | -4.54065641 | exon | down | 31,139,729 | 31,181,149 | Lncpint | 0.0060 | ENSMUSG00000044471.12 |
| chr6:89567587-89569412 | chr6 | 3.066062365 | exon | up | 89,567,587 | 89,569,412 | Chchd6 | 0.0045 | ENSMUSG00000030086.16 |
| chr7:116358636-116368189 | chr7 | -3.975433621 | exon | down | 116,358,636 | 116,368,189 | Pik3c2a | 0.0498 | ENSMUSG00000030660.9 |
| chr7:14578736-14609339 | chr7 | -4.018418838 | exon | down | 14,578,736 | 14,609,339 | Nlrp5-ps | 0.0428 | ENSMUSG00000041596.12 |
| chr7:46590607-46614776 | chr7 | -2.382004325 | exon | down | 46,590,607 | 46,614,776 | Sergef | 0.0370 | ENSMUSG00000030839.12 |
| chr7:59352999-59432670 | chr7 | 4.74985839 | intron | up | 59,352,999 | 59,432,670 | Ipw | 0.0148 | ENSMUSG00000100826.6 |
| chr7:59352999-59559862 | chr7 | 4.434242059 | intron | up | 59,352,999 | 59,559,862 | Lrrc28 | 0.0480 | ENSMUSG00000100826.6 |
| chr7:59955645-59956800 | chr7 | 1.280289411 | exon | up | 59,955,645 | 59,956,800 | Chd2 | 0.0005 | ENSMUSG00000100826.6 |
| chr7:67545366-67559969 | chr7 | 5.112538769 | exon | up | 67,545,366 | 67,559,969 | Nae1 | 0.0025 | ENSMUSG00000030556.13 |
| chr7:73499594-73503514 | chr7 | -4.207526213 | exon | down | 73,499,594 | 73,503,514 | Gatad2a | 0.0219 | ENSMUSG00000078671.11 |
| chr8:104516129-104518263 | chr8 | -4.686574372 | exon | down | 104,516,129 | 104,518,263 | Zfp827 | 0.0032 | ENSMUSG00000031878.18 |
| chr8:69935779-69936036 | chr8 | 2.65880786 | exon | up | 69,935,779 | 69,936,036 | Cyld | 0.0054 | ENSMUSG00000036180.15 |
| chr8:79091833-79092066 | chr8 | 1.518903844 | exon | up | 79,091,833 | 79,092,066 | Golga4 | 0.0327 | ENSMUSG00000071064.14 |
| chr8:88705226-88719396 | chr8 | -4.32248591 | exon | down | 88,705,226 | 88,709,996 | Zfp26 | 0.0142 | ENSMUSG00000036712.15 |
| chr9:118527010-118534705 | chr9 | 1.606271798 | exon | up | 118,527,010 | 118,534,705 | Sergef | 0.0136 | ENSMUSG00000038708.10 |
| chr9:20444259-20444985 | chr9 | 4.999919858 | exon | up | 20,444,259 | 20,444,985 | Ipw | 0.0049 | ENSMUSG00000063108.15 |
| chr9:29109396-29179254 | chr9 | -2.264516 | exon | down | 29,109,396 | 29,179,254 | Ntm | 0.0186 | ENSMUSG00000059974.10 |
| chr9:3441055-3450151 | chr9 | -4.295314737 | exon | down | 3,441,055 | 3,450,151 | Cwf19l2 | 0.0175 | ENSMUSG00000025898.6 |
| chr9:62068385-62088519 | chr9 | -1.073166578 | exon | down | 62,068,385 | 62,088,519 | Glce | 0.0284 | ENSMUSG00000032252.14 |
| chr9:72295279-72324154 | chr9 | -4.00412706 | exon | down | 72,295,279 | 72,324,154 | Zfp280d | 0.0464 | ENSMUSG00000038535.17 |
| chr9:72591751-72593360 | chr9 | 1.234346313 | exon | up | 72,591,751 | 72,593,360 | Rfx7 | 0.0015 | ENSMUSG00000037674.15 |
| chrM:2337-2427 | chrM | 1.817078635 | exon | up | 2,337 | 2,427 | None | 0.0200 | ENSMUSG00000064339.1 |
| chrM:2361-2427 | chrM | 1.994866588 | exon | up | 2,361 | 2,427 | None | 0.0180 | ENSMUSG00000064339.1 |
| chrM:428-523 | chrM | 2.253722132 | exon | up | 428 | 523 | None | 0.0000 | ENSMUSG00000064337.1 |
| chrM:4862-4947 | chrM | 5.496287899 | exon | up | 4,862 | 4,947 | ND2 | 0.0004 | ENSMUSG00000064345.1 |
| chrX:13093924-13103440 | chrX | 2.403801911 | exon | up | 13,093,924 | 13,103,440 | Usp9x | 0.0441 | ENSMUSG00000031010.17 |
| chrX:159311464-159332221 | chrX | -1.688893821 | exon | down | 159,311,464 | 159,332,221 | Rps6ka3 | 0.0338 | ENSMUSG00000031309.15 |
| chrX:18199158-18222877 | chrX | -4.217697339 | exon | down | 18,199,158 | 18,222,877 | Kdm6a | 0.0230 | ENSMUSG00000037369.17 |
| chrY:90760453-90844021 | chrY | 2.951743576 | intergenic | up | 90,760,453 | 90,844,021 | None | 0.0248 | n/a |

Table S6. In the comparison of PDST and PDCB, the top 20 differentially expressed circRNAs.

| CircRNA | Chromosome | Log_2_(fold change) | CircRNA_type | Regulation | CircRNA_start | CircRNA_end | Gene | *p*-value | Gene_id |
| --- | --- | --- | --- | --- | --- | --- | --- | --- | --- |
| chr10:90069472-90077142 | chr10 | -9.832354925 | exon | down | 90,069,472 | 90,077,142 | Anks1b | 1.62874E-15 | ENSMUSG00000058589.14 |
| chr11:12343744-12378359 | chr11 | 6.932148461 | exon | up | 12,343,744 | 12,378,359 | Cobl | 1.43288E-05 | ENSMUSG00000020173.17 |
| chr12:107989465-107989830 | chr12 | 7.219250201 | exon | up | 107,989,465 | 107,989,830 | Bcl11b | 2.48459E-06 | ENSMUSG00000048251.15 |
| chr12:38147503-38228123 | chr12 | 7.141368403 | exon | up | 107,989,465 | 38,228,123 | Dgkb | 4.24222E-06 | ENSMUSG00000036095.11 |
| chr14:103282598-103291362 | chr14 | 7.248454165 | exon | up | 103,282,598 | 103,291,362 | Mycbp2 | 3.08357E-06 | ENSMUSG00000033004.15 |
| chr14:16818690-16819151 | chr14 | 6.98358936 | exon | up | 16,818,690 | 16,819,151 | Rarb | 1.06902E-05 | ENSMUSG00000017491.9 |
| chr15:91786966-91814733 | chr15 | 7.788868538 | exon | up | 91,786,966 | 91,814,733 | Lrrk2 | 6.35358E-08 | ENSMUSG00000036273.15 |
| chr17:6137211-6139151 | chr17 | -8.963882346 | exon | down | 6,137,211 | 6,139,151 | Tulp4 | 0.021778696 | ENSMUSG00000034377.10 |
| chr19:50252534-50262367 | chr19 | 6.935961105 | exon | up | 50,252,534 | 50,262,367 | Sorcs1 | 2.29677E-05 | ENSMUSG00000043531.16 |
| chr1:39263319-39300733 | chr1 | 8.16492793 | exon | up | 39,263,319 | 39,300,733 | Npas2 | 4.87004E-09 | ENSMUSG00000026077.15 |
| chr4:124556045-124586127 | chr4 | 7.938285736 | intron | up | 124,556,045 | 124,586,127 | None | 2.05606E-08 | ENSMUSG00000098760.1 |
| chr4:23771757-23772887 | chr4 | 7.406511056 | intergenic | up | 23,771,757 | 23,772,887 | None | 1.08206E-06 | n/a |
| chr5:128268492-128269377 | chr5 | 7.508433335 | exon | up | 128,268,492 | 128,269,377 | Tmem132d | 5.01314E-07 | ENSMUSG00000034310.8 |
| chr6:54194026-54220702 | chr6 | -6.972705421 | exon | down | 54,194,026 | 54,220,702 | Chn2 | 3.92691E-07 | ENSMUSG00000004633.17 |
| chr6:54215662-54220702 | chr6 | -7.862452347 | exon | down | 54,215,662 | 54,220,702 | Chn2 | 1.52227E-09 | ENSMUSG00000004633.17 |
| chr6:99003200-99016665 | chr6 | 6.998444831 | exon | up | 99,003,200 | 99,016,665 | Foxp1 | 1.00061E-05 | ENSMUSG00000030067.17 |
| chr7:87596116-87603201 | chr7 | 6.918071423 | exon | up | 87,596,116 | 87,603,201 | Grm5 | 1.64584E-05 | ENSMUSG00000049583.15 |
| chr7:88074191-88075130 | chr7 | 7.015713664 | exon | up | 88,074,191 | 88,075,130 | Grm5 | 8.96526E-06 | ENSMUSG00000049583.15 |
| chr9:10168768-10172122 | chr9 | 7.541500086 | intron | up | 10,168,768 | 10,172,122 | Cntn5 | 5.01314E-07 | ENSMUSG00000039488.15 |

Table S7. In the comparison of PDST and PDHP, the top 20 differentially expressed circRNAs.

| CircRNA | Chromosome | Log_2_(fold change) | CircRNA_type | Regulation | CircRNA_start | CircRNA_end | Gene | *p*-value | Gene_id |
| --- | --- | --- | --- | --- | --- | --- | --- | --- | --- |
| chr13:94450953-94565200 | chr13 | 5.293897863 | exon | up | 94,450,953 | 94,565,200 | Ap3b1 | 0.0008 | ENSMUSG00000021686.6 |
| chr14:122133280-122139774 | chr14 | 4.949060556 | exon | up | 122,133,280 | 122,139,774 | Tm9sf2 | 0.0058 | ENSMUSG00000025544.13 |
| chr14:16818690-16819151 | chr14 | 5.84767022 | exon | up | 16,818,690 | 16,819,151 | Rarb | 0.00004 | ENSMUSG00000017491.9 |
| chr15:91786966-91814733 | chr15 | 6.654477579 | exon | up | 91,786,966 | 91,814,733 | Lrrk2 | 4.61135E-07 | ENSMUSG00000036273.15 |
| chr17:6457007-6624524 | chr17 | 7.215346006 | intergenic | up | 6,457,007 | 6,624,524 | None | 1.4068E-08 | n/a |
| chr18:33077993-33129833 | chr18 | 6.243258106 | exon | up | 33,077,993 | 33,129,833 | Camk4 | 6.93443E-06 | ENSMUSG00000038128.7 |
| chr19:28530873-28531986 | chr19 | -5.605020598 | exon | down | 28,530,873 | 28,531,986 | Glis3 | 0.0001 | ENSMUSG00000052942.13 |
| chr19:28530873-28540408 | chr19 | -4.764168448 | exon | down | 28,530,873 | 28,540,408 | Glis3 | 0.0070 | ENSMUSG00000052942.13 |
| chr1:154700371-154725920 | chr1 | -4.723570465 | exon | down | 154,700,371 | 154,725,920 | Cacna1e | 0.0084 | ENSMUSG00000004110.17 |
| chr1:190146952-190162313 | chr1 | -4.804492023 | exon | down | 190,146,952 | 190,162,313 | Prox1 | 0.0067 | ENSMUSG00000010175.13 |
| chr1:52489921-52490755 | chr1 | -4.978322734 | exon | down | 52,489,921 | 52,490,755 | Nab1 | 0.0033 | ENSMUSG00000002881.14 |
| chr3:58031207-58031793 | chr3 | 5.016622726 | intergenic | up | 58,031,207 | 58,031,793 | None | 0.003597137 | n/a |
| chr4:23771757-23772887 | chr4 | 6.276969569 | intergenic | up | 23,771,757 | 23,772,887 | None | 4.9666E-06 | n/a |
| chr5:138444752-138622988 | chr5 | -4.841134596 | intergenic | down | 138,444,752 | 138,622,988 | None | 0.005257584 | n/a |
| chr6:140543720-140563648 | chr6 | 4.980676469 | exon | up | 140,543,720 | 140,563,648 | Plekha5 | 0.003741889 | ENSMUSG00000030231.11 |
| chr6:37019916-37058028 | chr6 | 5.266343749 | exon | up | 37,019,916 | 37,058,028 | Dgki | 0.001018062 | ENSMUSG00000038665.15 |
| chr6:99003200-99016665 | chr6 | 5.863885631 | exon | up | 99,003,200 | 99,016,665 | Foxp1 | 3.85237E-05 | ENSMUSG00000030067.17 |
| chr8:77310731-77411128 | chr8 | 5.325073905 | exon | up | 77,310,731 | 77,411,128 | Arhgap10 | 0.000665549 | ENSMUSG00000037148.8 |
| chr9:22643745-22659145 | chr9 | 4.818756735 | exon | up | 22,643,745 | 22,659,145 | Bbs9 | 0.007513852 | ENSMUSG00000035919.16 |
| chr9:42655487-42675310 | chr9 | -4.769144013 | exon | down | 42,655,487 | 42,675,310 | Grik4 | 0.007694949 | ENSMUSG00000032017.14 |

Table S8. In the comparison of PDCB and PDHP, the top 20 differentially expressed circRNAs.

| CircRNA | Chromosome | Log_2_(fold change) | CircRNA_type | Regulation | CircRNA_start | CircRNA_end | Gene | *p*-value | Gene_id |
| --- | --- | --- | --- | --- | --- | --- | --- | --- | --- |
| chr10:7140319-7154485 | chr10 | 7.090184294 | exon | up | 7,140,319 | 7,154,485 | Cnksr3 | 7.30203E-09 | ENSMUSG00000015202.13 |
| chr10:90069472-90077142 | chr10 | 9.897243769 | exon | up | 90,069,472 | 90,077,142 | Anks1b | 1.32503E-16 | ENSMUSG00000058589.14 |
| chr12:107989465-107989830 | chr12 | -7.60997151 | exon | down | 107,989,465 | 107,989,830 | Bcl11b | 1.9036E-09 | ENSMUSG00000048251.15 |
| chr15:16823159-16832278 | chr15 | -6.655117805 | exon | down | 16,823,159 | 16,832,278 | Cdh9 | 9.65852E-07 | ENSMUSG00000025370.7 |
| chr15:5322244-5333050 | chr15 | -6.801975909 | intergenic | down | 5,322,244 | 5,333,050 | None | 3.80094E-07 | n/a |
| chr17:44638513-44639826 | chr17 | -7.368882842 | exon | down | 44,638,513 | 44,639,826 | Runx2 | 8.23963E-09 | ENSMUSG00000039153.17 |
| chr17:6137211-6139151 | chr17 | 9.031260355 | exon | up | 6,137,211 | 6,139,151 | Tulp4 | 0.020807801 | ENSMUSG00000034377.10 |
| chr19:28530873-28531986 | chr19 | -6.920326294 | exon | down | 28,530,873 | 28,531,986 | Glis3 | 2.11777E-07 | ENSMUSG00000052942.13 |
| chr1:140362949-140450304 | chr1 | -6.750648647 | exon | down | 140,362,949 | 140,450,304 | Kcnt2 | 5.075E-07 | ENSMUSG00000052726.15 |
| chr2:169883526-169886459 | chr2 | -7.326475403 | exon | down | 169,883,526 | 169,886,459 | Tshz2 | 1.3798E-08 | ENSMUSG00000047907.11 |
| chr3:29148347-29153601 | chr3 | -8.366801096 | exon | down | 29,148,347 | 29,153,601 | Egfem1 | -8.366801096 | ENSMUSG00000063600.14 |
| chr3:29148347-29170593 | chr3 | -6.868847481 | intron | down | 29,148,347 | 29,170,593 | Egfem1 | 2.26057E-07 | ENSMUSG00000063600.14 |
| chr4:124556045-124586127 | chr4 | -8.441585903 | intron | down | 124,556,045 | 124,586,127 | None | 6.69093E-12 | ENSMUSG00000098760.1 |
| chr5:128268492-128269377 | chr5 | -7.94043391 | exon | down | 128,268,492 | 128,269,377 | Tmem132d | 8.09924E-09 | ENSMUSG00000034310.8 |
| chr6:54194026-54220702 | chr6 | 7.043014619 | exon | up | 54,194,026 | 54,220,702 | Chn2 | 1.03392E-08 | ENSMUSG00000004633.17 |
| chr6:54215662-54220702 | chr6 | 7.929151545 | exon | up | 54,215,662 | 54,220,702 | Chn2 | 6.14273E-11 | ENSMUSG00000004633.17 |
| chr7:60785638-60856304 | chr7 | -6.688567137 | intergenic | down | 60,785,638 | 60,856,304 | None | 0.000353329 | n/a |
| chr9:123603727-123641914 | chr9 | -6.81034128 | intergenic | down | 123,603,727 | 123,641,914 | None | 4.67787E-07 | n/a |
| chr9:77164765-77190737 | chr9 | -7.432058445 | exon | down | 77,164,765 | 77,190,737 | Mlip | 6.11831E-09 | ENSMUSG00000032355.16 |

Table S9. Based on RNA-seq results and qRT-PCR results to show correlation across data.

| CircRNA | RNAseq- log_2_(Fold Change) | *p*-value | qRT-PCR log_2_(Fold Change) | *p*-value |
| --- | --- | --- | --- | --- |
| mmu_circRNA_0004144 | -3.85 | 0.036 | -0.71 | 0.02 |
| mmu_circRNA_0000468 | -3.78 | 0.045 | -0.64 | 0.038 |
| mmu_circRNA_0013321 | -3.86 | 0.034 | -0.75 | 0.006 |
| mmu_circRNA_0001320 | 1.07 | 0.043 | 0.43 | 0.048 |
| mmu_circ_0000870 | -2.29 | 0.026 | -1.00 | 0.031 |
| mmu_circ_0000517 | 3.68 | 0.01 | 1.72 | 0.02 |
| mmu_circ_0003292 | -3.37 | 2.8×10^-6^ | -3.70 | 0.001 |
